# Technological Approaches to Remote Monitoring of Elderly People in Cardiology: A Usability Perspective

**DOI:** 10.1155/2012/104561

**Published:** 2012-12-06

**Authors:** Susanna Spinsante, Roberto Antonicelli, Ilaria Mazzanti, Ennio Gambi

**Affiliations:** ^1^Department of Information Engineering, Marche Polytechnic University, Via Brecce Bianche 12, 60131 Ancona, Italy; ^2^Cardiology Operational Unit, INRCA Scientific Institute, Via della Montagnola 81, 60100 Ancona, Italy; ^3^University Clinic of Cardiology at Torrette Hospital, Marche Polytechnic University, Via Conca 71, Ancona, Italy

## Abstract

Moving from the experience gained in home telemonitoring of elderly patients with Congestive Heart Failure, that confirmed a reduction of the rehospitalization rate and an improved monitoring of drugs assumption by the patients, this paper extends the evaluation of technological approaches for remote health monitoring of older adults. Focus of the evaluation is on telemedicine effectiveness and usability, either from a patient's or a medical operator's perspective. The evaluation has been performed by testing three remote health platforms designed according to different technological approaches, in a realistic scenario involving older adults and medical operators (doctors and nurses). The aim of the testing activity was not to benchmark a specific solution with respect to the others, but to evaluate the main positive and negative issues related to the system and service design philosophy each solution was built upon. Though preliminary, the results discussed in the paper can be used as a set of guidelines in the selection of proper technological equipments for services targeted to elderly users, from a usability perspective. These results need to be complemented with more focused discussions of the ethical, medical, and legal aspects of the use of technology in remote healthcare.

## 1. Introduction

The impact of ICT (Information and Communication Technology) on the healthcare sector is a double-face phenomenon. On one hand, many effective and significant advances are continuously taking place, especially in the field of medical treatments, and devices designed for their delivery. Also the management of medical data and patients' records is experiencing a kind of digital revolution; thanks to the widespread introduction of the electronic health record to gradually replace traditionally heterogeneous, and often partially hand-processed, data management services. On the other hand, however, although appropriate technology is available, a very limited spreading of remote health monitoring solutions is evidenced, especially among those users that could really benefit from it, such as elderly people or physically disabled people.

Since several years, research studies and projections show that most of the developed countries are experiencing a demographic shift [1]; as an example, the life expectancy for males and females in Europe has increased from 45.7 and 49.6 to 75.0 and 79.9 years, respectively, in less than a century. Looking at long-term projections, the process of ageing is set to increase at an even faster pace; moreover, there is a growing concern about the low birth rate in industrialized countries. Demographic changes affect a wide range of economic and social fields, as well as policies concerned with health, social welfare, housing, and many other issues. Basically, most of the available research reports and studies claim that, despite a necessary significant financial investment at a first stage, the adoption of ICT in the delivery of remote health services among population can really allow strong resource savings. As a matter of fact, population ageing will lead to an increase in the proportion of the population with disabilities or chronic illnesses, that will lead to increasing expenses to cope with, by healthcare systems and social welfare, in general. With the adoption of ICT and related services [3], elderly or disabled people will be able to stay in their home environment while being medically supervised and possibly treated. This is of particular importance for older people, as the prevalence of chronic diseases generally increases with growing age.

National systems of social care will be confronted with challenges regarding the *income side*, as demographic ageing means that the number of older people is growing, while the share of those of working age (15–64 years) is decreasing. The demand for health services and social welfare will considerably increase in coming years: this will yield problems in financing the social benefits, under the corresponding social security systems. Within European national healthcare systems, due to the needs for assistance of elderly people, most member states spend currently between 30 and 40% of total health expenditures on elderly persons (i.e., those aged over 65), as well as they are making expenditures on long-term care for the elderly. Given this scenario, the widespread adoption of health provisioning solutions based on ICT becomes unavoidable. Technology may help in limiting the impact of costs faced to provide social and medical services: some assistance requests may be solved, for example, without moving health operators, by establishing a direct and “live” communication session between doctors and patients. With monitoring and data transmission both occurring daily, patients might be able to avoid numerous trips to a physician's surgery, and physicians could quickly act and tailor medical treatments to variations of the patient's health condition. Despite the well-recognized positive effects that a widerspread adoption of telemedicine could bring, not so many legacy systems are already operating, especially in Italy. Several pilot and experimental initiatives have been carried on [4], but it is possible to say that a standard adoption of telemedicine, as a way of delivering health services, is still lacking.

Previous experiences, by some of the authors, in telemedicine for the home monitoring of elderly patients with Congestive Heart Failure (CHF) [5–7] provided positive outcomes, in terms of reduction of the rehospitalization rate, and improvements in the regular assumption of drugs and medications by the patients. Moving from this background, the paper discusses the preliminary results of an experimental study concerned with the evaluation of different kinds of technological platforms for the delivery of remote healthcare services to elderly people. The target users selected for the study may feature a number of possible health-related problems, but the telemedicine solutions were chosen with a focus on cardiology-related diseases. Comorbidity in geriatric population poses strong limitations in the patients' lifestyle and may greatly reduce their physical or mental independence: this motivates the need of evaluating a technological platform designed for remote health monitoring under strict usability and accessibility criteria [8–10]. The ideal telemedicine system should enable users, even older ones, the ability to easily self-monitor various health parameters, and provide important information to medical operators, thus facilitating timely healthcare decisions [2, 11].

The purpose of the present study is to assess different technological approaches to the design of remote health-monitoring platforms, with respect to specific requirements that are defined according to the target elderly patients. The comparison is based on three sample solutions, but the outcomes herein discussed are intended as general ones and not referred to a specific system. Because of the low number of users that it has been possible to involve in the experiment up to now, at this time the data provided can be considered as a pilot study for the validation of telemedicine solutions for the elderly.

## 2. Materials

### 2.1. Subjects

The expected users for the services and systems tested in the experiment are older adults of age ≥65 years. The target diseases to monitor are heart-related pathologies, with a specific focus on heart failure (HF); the availability of several biomedical sensors, such as the ECG monitor, makes it possible to extend the monitoring activity also to other pathologies, such as atrial fibrillation, arrhythmia, and suspected heart-attack related symptoms. In any case, due to the comorbidity that typically affects elderly patients, it is expected that a number of physical and/or cognitive impairments could prevent the patients from efficiently performing some basic operations needed to interact with the technological platforms. As a consequence of this quite common condition, it is foreseen to test the usage of telemedicine systems also by caregivers or nurses, that are often in charge of the older patient's care. The experimental tests performed up to now on the available systems involved three older adults at their homes (one of them was supported by a caregiver, the other two were independent), and a professional nurse in charge of a residential structure for retired elderly people. From the operators' side, the experiment involves a couple of students approaching their degree (a professional nurse and a medical doctor, resp.), a cardiology specialist, acting as supervisor and dealing with the clinical evaluation of the health data collected, and an electronic engineer dealing with the technical issues related to the proper set up and configuration of the systems tested.

### 2.2. Monitoring Systems

 Three sample telemedicine systems have been adopted for the experimental tests. As previously stated, the focus of this work is not on benchmarking a specific system with respect to the others, but on evaluating different design approaches, such as the architecture upon which the solution is built, the kind of devices and connection techniques used, and the way according to which data are collected and made available to the medical operators.

The systems under evaluation will be referred to as *System A*, *System B*, and *System C*.

*System A* was originally conceived for adoption in emergency conditions. The system is designed to be portable: all the medical devices used to collect the patient's vital parameters (blood pressure monitor, ECG device, weight scale, pulse oximeter, expiratory flow meter, stethoscope, and blood glucose monitor), together with the central box they shall be connected to, are stored within a watertight case. The case also contains the power supply unit, that needs a grid plug to work, and a video monitor integrated into the upper case side. When connected to the central box, that runs a Linux-based OS, the medical devices allow remote monitoring of a patient's vital signs, as and when required. Measurement results are stored in a central database, reached through a wireless data connection supplied over UMTS/HSDPA. The box is able to set up a video-communication session over a wireless data link, between the patient and the remote medical center, and to manage the exchange of biomedical data between them, during the video session. Medical devices may be connected to the box through Bluetooth, Infrared technology, or via USB. For the connection of the stethoscope, a custom audio cable is provided, that allows to transfer the audio signal captured by the stethoscope to the remote medical operator, over the same audio channel set up during the A/V communication session.

*System B* is designed according to a similar concept, that is, a set of medical devices connected to a central box. In this case the box is conceived to be a desktop unit, that is expected to work in a home environment, once located in a fixed position. The box is actually provided through a touchscreen-enabled desktop PC unit, running the Microsoft Windows XP O.S., and the patient interacts with the system only by means of the touchscreen interface. The central unit may be connected to the remote health center either over a wired connection (xDSL, Ethernet) or a wireless one (UMTS/HSDPA, Wi-Fi). A video communication session may be established through the system, exploiting an integrated webcam and the touchscreen monitor. The set of medical devices is the same of *System A*, and a Bluetooth enabled stethoscope is also supported, for the remote patient auscultation.

*System C* features a mobile-oriented approach: the central unit, to which the different medical sensors may connect, becomes a portable device, such as a mobile phone or a tablet PC running Android O.S., acting as a gateway between each biomedical sensor and a remote data repository. The repository is accessed by the mobile client in a secure way through a wireless broadband connection established over UMTS/HSDPA or Wi-Fi. The gateway can interact with the same devices of *System A* only over Bluetooth links, with the exception of the stethoscope that is not supported. The medical operator is enabled to access a remote web platform that interfaces the data repository; a video communication session may be set up from the patient to the remote healthcare center, through the remote web platform acting as a bridge.

Table 1 provides a summary of the systems' description, with respect to the technologies adopted.

## 3. Methods

### 3.1. System Rating Criteria

 Dealing with technological solutions aimed at enabling the user (i.e., the patient) to self-manage the collection of his health data, for their analysis and evaluation by a remote medical operator, different rating criteria shall be defined. Besides a technical evaluation, based on the features provided by each system, that can be tested and rated according to quantitative figures, it is necessary to provide also a user-related evaluation, that can be further itemized into a patient's perspective and an operator's perspective.

#### 3.1.1. Technical Rating Issues

 A telemedicine system is typically obtained by integrating different technologies and subsystems, each of them covering specific functionalities needed to provide the requested services. In this case, the focus is on remote monitoring of health-related parameters, that may help in preventing or limiting the impact of heart failures and related symptoms. According to the short description of the systems under test, provided in the previous Section, it is possible to identify some common blocks composing the architectures herein considered as follows:

- client side: the set of biomedical sensors used to monitor the patient's parameters;
- client side: the central unit acting as a gateway among the patient's premise and the remote health center;
- server side: a remote platform including a data repository and a software interface to access it and to perform the requested data analyses.

Each of these blocks may provide specific functionalities, with different degrees of performance. As already stated, the *set of biomedical sensors* used by *System A*, *B*, and *C* is the same, apart from the stethoscope device, that is not supported by *System C*. Each sensor is available in different flavors, that is, with a wired or wireless connection to the central unit, and the selection of the specific sensor to use shall be based on technical issues (the possibility of integrating or interfacing the sensor to the client central unit), but also on usability-related aspects. With the aim of defining general rating criteria, that can fit almost any sensor device, and due to the target users the system should support (elderly or partially disabled patients), it is expected to assign higher scores to those solutions that integrate wireless sensors, instead of wired ones; sensors that are able to provide a visual or acoustic feedback about the proper data acquisition and transfer to the central unit; sensors that notify their battery power level; sensors that do not require an initial configuration by the user, or at least, that do minimize the number of configuration steps necessary before collecting the desired data. Assuming a rating parameter defined as *s* and a scale from 5 (highest rate) to 1 (lowest rate), the rating criteria for each sensor the system is equipped with, may be defined according to Table 2. By scoring each device in the set of sensors, it is possible to get a global score for the system sensor set, named *S*, so that

(1) $$\begin{array}{c} S\equiv \sum_{j=1}^{N}\left(\sum_{i=1}^{M}s_{i}^{j}\right), \end{array}$$

where *N* is the number of sensors in the set, *M* is the number of sensor rating issues (*M* = 4 in our model), and *s*_*i*_^*j*^ is the score assigned to each *i*th rating issue, for each *j*th sensor in the set. The *S* parameter is not normalized, in order to properly account for the different number of sensors each telemedicine system may provide.

About the *central unit* at the client side, that is in charge of collecting the sensors' data and interfacing the patient to the remote healthcare center, it is possible to identify a number of features for its technical rating. The features are selected to be applicable to different solutions, but, at the same time, they are enough detailed to allow for a meaningful rating process. According to the amount and nature of the data the unit has to manage and transfer to the remote center, and one of its basic feature is the network interface the unit is equipped with. Assuming that an “ideal” solution should enable the joint transmission of audio, video, and data streams, corresponding to the information flows that take place during a remote, but “live” medical check, where a doctor is able to interact with the patient in real time, the highest score should be assigned to systems supporting broadband network connections, and the lowest score should be given to systems provided with narrowband interfaces. On the other hand, assuming the technological platform may be decomposed into “atomic” services (e.g., the transmission of the blood pressure data only, or the transmission of the ECG data only) a narrowband connection could be also accepted, but this would limit the potential benefits obtainable from the platform itself, as a whole.

The flow of information, during a data collection session with no active video communication, is basically asymmetric, with the client side mainly uploading data towards the remote repository. However, the amount of data is limited, so that we assume that symmetric or asymmetric connections may be rated the same score. Besides that, it is important also to check if the network interface supported is a wired or a wireless one. This scoring criterion, however, also depends on the target users the system is designed for. Limiting the present discussion to systems designed for the elderly or partially disabled people, it is assumed to provide higher scores to wireless solutions, because they result less invasive with respect to the patient's home environment and do not require any cabling operations to be set up. To this aim, we assume that the initial set up of the client central unit wireless connection is provided by technicians involved in the service delivery. The performance of the data transfer process may be optimized if some audio and video compression techniques are applied: the client central unit should be designed in order to trade off compression efficiency and audio/video quality. Highest score will be given to systems providing compressed video at CIF resolution (355 × 288 pixels) as a minimum and compressed audio at a tunable quality (the highest one required when transmitting the patient's auscultation audio signals). Another feature that may be critical to the effective use of the telemedicine system is the possibility offered by the client central unit of automatically switching between synchronous and asynchronous data management: the former enables a real time data transmission to the remote center, and the latter allows for a temporary storage of the collected data within the unit itself, and a delayed transmission, as soon as the network connection becomes available. This feature is a basic one if the central unit adopts a wireless network interface, in order to avoid data losses due to temporary connection outages. In order to minimize the user's actions on the system, to configure the central unit or to update its software components, it is expected to assign higher scores to solutions supporting a remote client management, that provides automatic software updates and configurations. Finally, the evaluation will assess positively the solutions ensuring data privacy and security, by means of protected data connections established between the patient and the remote healthcare center. The technical rating criteria defined for the client central unit are summarized in Table 3. Again, it is possible to define a central unit global score, named CU, as

(2) $$\begin{array}{c} \text{CU}\equiv \sum_{k=1}^{K}{\text{cu}}_{k}, \end{array}$$

where *K* is the number of rating criteria (*K* = 7, in our model), and cu_*k*_ is the score assigned to each criterion.

The *server side* evaluation of the telemedicine system typically involves two basic components: a data repository, where the data collected by the remote patients are stored, and a software interface, that allows the operators to access the data and to perform the requested evaluation. About the data repository, the recent evolutions in the field of cloud-based data management [12, 13] suggest the adoption of the SaaS (Software as a Service) paradigm, instead of the more traditional PaaS (Platform as a Service) one. The former ensures higher reliability, because data are not physically located in a machine but are spread across a cloud of nodes, cooperating towards the provision of a specific service. As a consequence, once defined a rating parameter rp referred to the remote platform composing elements, it is assumed to assign the highest score (rp = 5) to a telemedicine solution that relies on a SaaS paradigm, a medium score (rp = 3) to a solution adopting a PaaS approach, and the lowest score (rp = 1) to solutions requiring a dedicated local storage server.

Given the specific nature of the data collected by the remote clients and transferred to the repository, a number of security services shall be ensured for any service provisioned through the remote platform, such as data integrity protection, by the adoption of secure transmission protocols and certificates between the client gateway and the remote platform; separation of legally valued and identity-related data; safe management of the remote platform within the data center adopted (backup policies, continuous monitoring, and system access control); access control and tracking, with specific focus on the personal patients' data; authenticated and controlled accounts; security policies for passwords management. Different scores may be assigned to each issue, according to the specific security solution adopted, its strength and robustness.

About the technical features related to the software interface used by the operators to access the set of data collected by each patient, highest rate is assigned to solutions providing a web-enabled access, so that the operator may perform the requested evaluation irrespective of his physical location (mobility support), simply by resorting to a web browsing application. Software interfaces designed for multimodal and multichannel access (i.e., by a PC or a mobile device) are preferable to solutions requiring specific plugins or software components to be installed, or executed on specific OSs. Finally, the implementation of security services for access authentication and tracking is another issue, ranked according to the robustness of the specific solution adopted. The set of ranking criteria defined for the remote platform is summarized in Table 4. As previously done, a global remote platform score, named RP, may be defined as

(3) $$\begin{array}{c} \text{RP}=\sum_{z=1}^{R}\text{r}{\text{p}}_{z}, \end{array}$$

where *R* is the number of ranking criteria adopted (*R* = 10 in our model), and rp_*z*_ is the score assigned to each ranking issue.

Once the technical rating criteria for each subsystem have been identified, and the corresponding rating parameter defined, it is possible to derive a global system rating parameter, referred to the technical features of the telemedicine solution as a whole, as

(4) $$\begin{array}{c} \text{TTP}=\alpha \cdot S+\beta \cdot \text{CU}+\gamma \cdot \text{RP}, \end{array}$$

where TTP is the *technical telemedicine system parameter*, *α*, *β*, and *γ* are weighting factors used to differentiate the impact of each subsystem on the global technical rating of the telemedicine solution. We assume to have {*α*, *β*, *γ*} ∈ [0; 1]. The value assigned to each weighting factor during the assessment process depends on the expected or estimated impact of each sub-system on the effectiveness, reliability, and performance of the whole system. As an example, if the telemedicine solution is expected to provide a second-opinion service among health operators, the weighting factors will satisfy the following condition: *α* < *β* ≪ *γ*. On the other hand, a telemedicine system designed to provide remote healthcare services in underserved areas (such as rural areas) will feature a different condition, like: *α*≅*β* and {*α*, *β*} > *γ*. The specific value assigned to each weighting factor is consequently related to the service model implemented through the telemedicine system.

#### 3.1.2. User-Related Rating Issues: Patient Side

 The definition of suitable rating criteria from a patient's perspective is a critical point to the proper evaluation of the global telemedicine solution. As a matter of fact, it is not possible to completely generalize the definition of such criteria, as they are strictly dependent on the target users the system is designed for.

This work is focused on elderly or partially disabled people, so it is mandatory to account for their specific needs, related to the variety of physical or cognitive impairments that may characterize the patient's condition. In the case of elderly patients, several rating criteria may be defined to assess the telemedicine system from a usability perspective. Among them, we consider the ergonomics of the medical devices the patient should use to collect his health data (not too small dimensions, effective visual or acoustic signalling, and easy switch on/off); suitably formatted and delivered instructions to guide the user; easy and quick switch on/off of the client central unit; clearness and easiness-of-use of the Graphic User Interface (GUI) the client central unit is equipped with; reliability of the application used to manage the sensor devices and to collect the patient's data (e.g., robustness against user's misbehaviour or unexpected commands, automatic restart of the application in case of unexpected crashes, and robustness against lacking data connection); cognitive support and feedback messages provided by the application to the user, during the execution of different tasks; user's satisfaction, given by the rate of successful execution of the desired task. The rating of these criteria is not as straightforward as the previous ones, so it is assumed to provide a score that may vary from a minimum value of 1 (the criterion is completely not satisfied) to a maximum value of 10 (the criterion is completely satisfied). The assessment of a specific system from the patient's perspective may be performed through the submission of a questionnaire to the user, asking to rate each single issue.

#### 3.1.3. User-Related Rating Issues: Operator Side

 The operator's perspective in the evaluation of a telemedicine system basically deals with the impact of the implementation on the work processes, that are typically well defined and standardized. Assuming that the service model related to the provisioning of remote health assistance has been defined, the operator will typically assess the effectiveness of the telemedicine system, intended as the set of available functionalities and their performance. The way according to which patients' personal records and health data are organized and made accessible through the software interface, the available tools to search and select specific data, and the design of the GUI are critical to the successful acceptance of the telemedicine system as a “real” diagnostic procedure [14]. In this sense, it is very important to have the possibility of customizing some of the interface features, according to the requirements expressed by the medical operators, derived from the daily clinical practice. As a consequence, one of the ranking criteria that may be defined deals with the degree of customization that may be allowed by the system design. The easiness-of-use, the clear organization of the information provided by the system interface, and the availability of specific planning tools (such as online agendas, shared folders, and collaborative management of data certification processes) may be ranked according to a score varying in a range of values, as for the patient's evaluation scale.

Table 5 summarizes the patient's and operator's rating criteria discussed above.

### 3.2. Measurements

 Measurements may be provided about the system technical functionalities and performance. They support an objective evaluation of a telemedicine solution and its reliability. A first issue that is taken into account is the agreement between the data collected by means of the telemedicine system and the data collected in a traditional way (i.e., by a medical operator). This is a basic issue upon which the validity of the remote health monitoring service relies. In this work, given the application focus on heart-related diseases, the reliability of the ECG and blood pressure measurements has been evaluated by the contemporary acquisition of these parameters through a standard modality and through the platform. Other issues that have been considered are the average number of attempts requested to establish a connection between the patient and the remote health center, the incidence of connection losses during a remote health examination, the incidence of malfunctions or crashes during parameters' acquisition by the patient, and the amount of lost or corrupted (not readable) measurements. Among the other elements that may be evaluated, even if in a not strictly quantitative way, it is possible to cite the stability of the audio/video communication session, the quality of the audio and video signals, and the effectiveness of the audio session in enabling a remote heart and pulmonary auscultation of the patient.

## 4. Results and Discussion

### 4.1. Technical Evaluation

**Figure 1:**
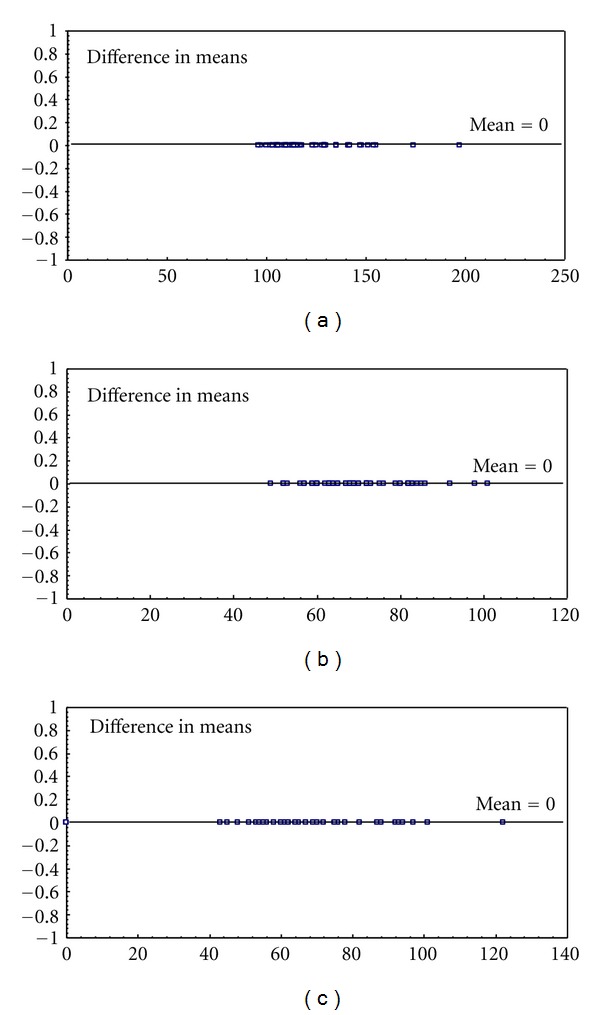
Bland-Altman plots comparing the agreement between traditional and telemedicine-based acquisition of: (a) sistolic blood pressure, (b) diastolic blood pressure, and (c) heart rate values (*System C*).

 The available telemedicine systems, *A*, *B*, and *C*, have been tested first with respect to the agreement of the ECG measurements and blood pressure values collected by the platform or by the traditional procedure. All the systems tested have provided corresponding values of the diastolic and sistolic blood pressure and of the heart rate estimated by the blood pressure monitor. Figures 1(a), 1(b), and 1(c) show the Bland-Altman plots obtained by comparing the traditional methodology (parameters collected by the medical operator: nurse or doctor) to the telemedicine-based methodology, over 52 measurement sessions, respectively, for the sistolic, the diastolic blood pressure and for the heart rate value.

**Figure 2:**
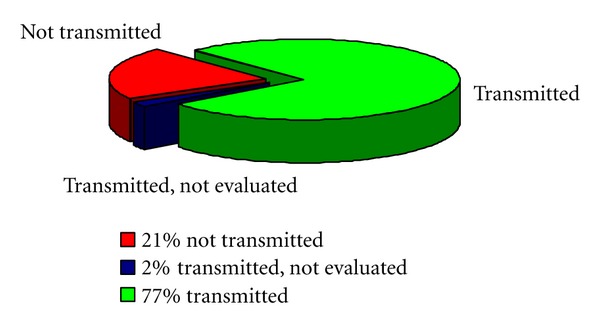
Percent amounts of correctly received ECG patterns, not received ECG patterns, and ECG patterns that were received but could not be evaluated, over 52 monitoring sessions (*System C*).

About the correct acquisition of ECG patterns, different performances have been obtained by the three systems. In the case of *System A* and *System C*, both adopting a wireless broadband connection on UMTS/HSDPA links, some ECG patterns that were correctly transmitted from the ECG monitor device to the client central unit, did not arrive to the remote repository, reasonably due to problems during transmission over the WAN radio connection.  Figure 2 refers to *System C* and shows that over a set of 52 collected ECG patterns, 21% of them were not transmitted, 77% of them were correctly transmitted to the remote repository, and 2% of them were correctly received but could not be evaluated by the cardiology specialist, due to the complexity of the ECG patterns related to specific heart diseases. It is interesting to point out that 26 monitoring sessions over 52 used a mobile phone as the gateway (i.e., the client central unit) and 26 monitoring sessions used a tablet. Missing ECG patterns are to be referred only to the monitoring sessions performed with a tablet (42.3% over 26 sessions). Among the 42.3% missing transmissions, 81.8% may be referred to connection problems, but 18.2% are to be referred to erroneous use of the device by the operator. This result, though preliminary, evidences the basic role of operators or patients in correctly handling the medical equipments and the telemedicine systems to ensure the effectiveness of the process.

*System B*, that uses a wired network connection, should not exhibit missing transmission events. However, we found that if the ECG acquisition is performed offline (e.g., when the network connection is not available, or the client central unit is not connected), the ECG patterns are not always transferred to the repository once the system goes online again. Of course, this issue is to be solved to avoid missing data when the patient or the operator performs the measurements. In similar conditions (lacking network connection), *System A* did not work properly, whereas *System C* was able to store the data collected by the user, for a successful later transmission to the repository.

**Figure 3:**
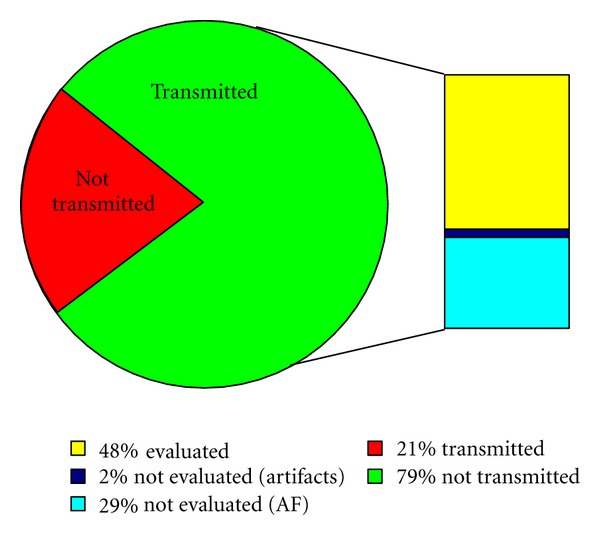
Percent amounts of ECG patterns that were correctly received but could not be evaluated.

With respect to the proper acquisition of ECG patterns, it is necessary to point out that, even when an ECG measurement is correctly transferred to the remote repository, it is possible that the ECG pattern may not be evaluated by the cardiology specialist. This is the case for ECG patterns in which possible artifacts (due to the acquisition or to the transmission process) modify the ECG curve and make it not useful to evaluate some heart diseases (like those determining low voltage values in the ECG signal). In the case of patients suffering from Atrial Fibrillation (AF), this condition may avoid the acquisition of useful ECG patterns.  Figure 3 provides details about the incidence of these situations on the 52 measurement sessions performed, when looking at the *P* curve amplitude and the *PR* interval duration.

**Figure 4:**
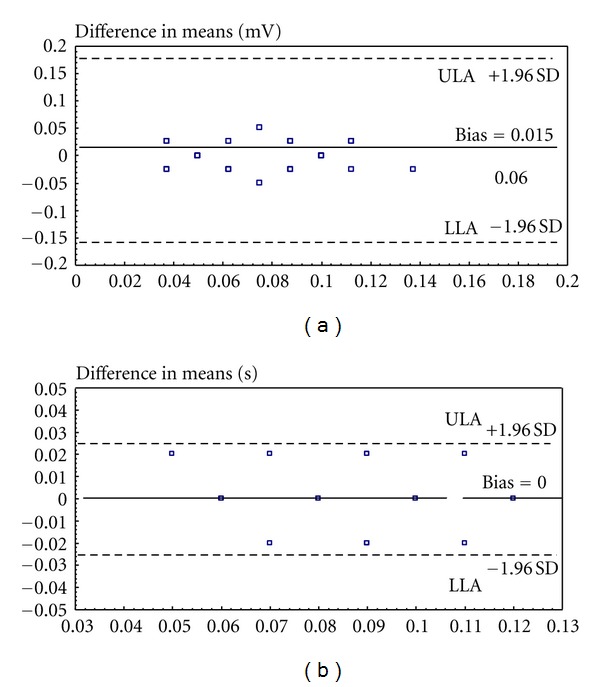
Bland-Altman plots comparing the agreement between traditional and telemedicine-based estimation of (a) *P* curve amplitude and (b) *PR* interval duration, from DI derivation (ULA: upper limit of agreement, LLA: lower limit of agreement).

For the ECG patterns that have been evaluated, Bland-Altman plots of Figures 4(a) and 4(b) show the agreement of the traditional and the telemedicine-based methodologies in the estimation of the *P* curve amplitude and the *PR* interval duration, from DI derivation. The same agreement is confirmed also for the *PR* interval and the *QRS* complex, from different derivations. The only significant remark about the analysis of ECG patterns collected by the telemedicine system is the difficulty of evaluating patterns in which the ECG signal amplitude is reduced (<0.5 mV), due to an increased amount of artifacts, with respect to ECG patterns obtained by the traditional methodology, under the same conditions.

Looking at the performance of all the systems tested, in terms of ECG and blood pressure-related parameters, we may state that if a telemedicine solution relies on a wireless network connection, it is of primary importance to ensure an effective storage capability for the parameters collected when the connection is lacking. Assuming the wireless connection is reliable, it is necessary to pay maximal attention to the parameter acquisition process (especially the ECG measurement), in order to minimize the impact of source artifacts (e.g., due to patient's baseline wander). From a technical point of view, the telemedicine system should possibly apply some coding techniques to improve the signal robustness against errors due to the channel (at the transmitter side) and suitable techniques at the receiverto recover possibly missing data due to link outages (e.g., signal concealment techniques).

In order to clarify how the proposed methodology may help in comparing different technological platforms, with respect to a specific service requested by a patient, Table 6 shows the proposed rating criteria collected in a features chart, together with a requirements definition that may be representative of the service under consideration. Similar charts can be arranged for different services, by resorting to the set of ranking criteria previously defined. By checking a given platform versus the features chart corresponding to the requested service, it is possible to estimate if the platform adheres to the expected capabilities or not. In the event several platforms comply with the requirements expressed by a features chart, it will be possible to select the best platform by comparing the level of requirement compliance provided (e.g., the platform that provides the greatest local storage capability should be preferred over the other ones).

### 4.2. Test Use Case

 The three systems available for experimental purposes have been tested towards the same target service, that is, to provide a comprehensive healthcare service to older adults. Such a service includes heart monitoring, blood pressure monitoring, weight monitoring, and A/V connection capability to possibly perform real time remote auscultation through the stethoscope. The ranking criteria introduced for sensors, central unit, and remote platform evaluation were applied, to objectively evaluate the available systems. Looking at (4), it is possible to say that irrespective of the values assigned to *α*, *β*, and *γ*, these weighting can be the same for *System A*, *B*, and *C*, as we are considering the same target service. For the specific use case under evaluation, the weighting factors were set as *α* = 1, *β* = 0.8, and *γ* = 0.6, motivated by the fact that the technical features of sensors and central unit have a stronger impact on older users than the technical features of the remote platform. The numerical figures obtained for the three systems under test by applying the technical evaluation criteria introduced in Tables 2, 3, and 4 are summarized in Table 7.

The objective technical evaluation provides the highest score for *System C*, followed by *System B* and *System A*. Looking at Table 7, we may say that *Systems A* and *B* provide similar technical performance about the client central unit, better than *System C*; *System B* features the best set of sensors among the solutions tested, whereas *System C* provides the best performance for its remote platform. The objective evaluation results confirmed the opinions provided by the users, and also the expectations expressed by the medical operators. An ideal optimized solution should include the best features revealed by the analysis of each system.

About the other technical features that may be evaluated according to the ranking criteria introduced, it is possible to provide general remarks, according to which

- *System A* featured the highest number of attempts necessary to establish a connection, followed by *System C*. In some conditions, also *System B* required several attempts, even if equipped with a wired interface;
- the incidence of connection losses, and system malfunctions or crashes, was the highest for *System A*, followed by *System B*. *System C* is the most robust against link outages, with a satisfactory storage capability of collected data at the client gateway;
- *System B* provided the best quality for the audio/video sessions, the strongest stability of the connection, and the best performance in remote patient auscultation (especially the pulmonary one). It is possible to say that, reasonably, a wired connection, when properly established, may ensure better real time multimedia sessions between the patient and the remote operator.

### 4.3. Usability Evaluation

 Due to the limited number of patients that could test the available telemedicine platforms, up to now, it is possible to provide only a general overview of the usability issues that were evidenced during the experimental activities. Dealing with elderly or partially impaired people, one of the main barriers to the effective empowerment of the user is given by the ergonomics of the devices composing the telemedicine platforms. Sensors are typically small and usually have small displays to provide feedback information about the collected data. The client central unit should be designed to have easy-to-handle I/O interfaces: with respect to this issue, *System B* probably represents the best solution, given its quite big touchscreen monitor, whereas *System A* represents the most critical one, due to its small remote control and to the number of different steps that need to be performed by pressing one or more buttons, before activating the service. These issues joint the need of a clear and intuitive GUI expressed by the users and highlight the need of a specific technological design suitable for elderly or impaired people, by taking into account their requirements, either physical or cognitive ones. *Systems B* and *C* provided the highest rates in patient's satisfaction when performing a specific task, with *System B* also assessed first with respect to the easy-to-use GUI, and *System C* ranked first for its quick switching on/off.

From the operators' perspective, the three systems provided a satisfactory set of sensors, apart from *System C* that does not yet support a wireless stethoscope; it is actually very important for the followup of specific heart-related diseases, like CHF. The operators were particularly interested in the possibility of customizing the structure and the organization of the software interface used to access the remote platform and the data repository. Critical issues, from the operators' point of view, are the possibility of establishing real time audio/video communication sessions with a patient or a caregiver and the mobility support provided by the telemedicine system, that makes it possible to assist the remote patients even out of the health center premises.

## 5. Conclusion

 This paper presented a possible methodology for the technical and usability-related evaluation of three remote health platforms, designed according to different technological approaches in a realistic scenario involving older adults and medical operators. The aim of the testing activity was not to benchmark a specific solution with respect to the others, but to evaluate the main positive and negative issues related to the system and service design philosophy each solution was built upon. The preliminary results presented by the paper may represent a set of guidelines in the selection of proper technological equipments for services targeted to elderly users. The evaluation activity is currently ongoing, in order to get statistically significant figures and to come to the definition of an objective procedure for the evaluation of telemedicine solutions.

## Figures and Tables

**Table 1:** Systems technology summary.

	*System A *	*System B *	*System C *
System topology	Centralized	Centralized	Centralized
System use	Portable	Residential	Mobile
OS	Linux based	Windows XP	Android
Power supply	Grid	Grid	Battery
Central unit	PC-based box	PC-based desktop	Mobile phone/tablet
Data connection	WAN	xDSL/WLAN/WAN	WAN/WLAN
Device connection	Bluetooth, USB	Bluetooth, USB	Bluetooth
User I/O interfaces	Remote control video, audio	Touchscreen video, audio	Touchscreen video, audio
Audio/video communication	Yes	Yes	Yes

**Table 2:** Technical rating criteria: sensors.

Issue *i*th	Rating *s* = 5	Rating *s* = 1
(1) Connection	Wireless	Wired
(2) Feedback on data acquisition	Acoustic + visual	No feedback
(3) Battery life status	Notified	Not notified
(4) Configuration steps	Not needed	More than 1 step

**Table 3:** Technical rating criteria: client central unit.

Issue *k*th	Rating cu = 5	Rating cu = 1
(1) Network interface	Broadband	Narrowband
(2) Network connection	Wireless	Wired
(3) Video compression	Yes, resolution ≥ CIF	Yes, resolution < CIF
(4) Audio compression	Yes, tunable	Yes, not tunable
(5) Data management	Synchronous and asynchronous	Only synchronous, or only asynchronous
(6) Remote system management	Yes	No
(7) Data privacy and security	Yes	No

**Table 4:** Technical rating criteria: remote platform.

Issue *z*th	Rating rp = 5	Rating rp = 3	Rating rp = 1
(1) Platform paradigm	SaaS	PaaS	Dedicated storage server
(2) Data integrity protection	Strong	Weak	No
(3) Data separation	Yes	//	No
(4) Safety management	Strong	Weak	No
(5) Data access control and tracking	Strong	Weak	No
(6) Authenticated accounts management	Yes	//	No
(7) Secure password management	Strong	Weak	No
(8) Web enabled access	Yes	//	No
(9) Multimodal and multichannel support	Yes	Partial	No
(10) Authenticated access	Strong	Weak	No

**Table 5:** User rating criteria: patient and operator sides (score in [1, 2]).

Patient's side	Operator's side
Ergonomics of the medical devices	Available functionalities and tools
Suitably formatted instructions	Data management and organization
Quick switch on/off	GUI design
Easy-to-use GUI	Possible customization
Software reliability and robustness	Easy-to-use GUI and tools
Cognitive feedback	Intuitive functions and design
Satisfaction in task execution	Availability of planning tools

**Table 6:** Sample platform features chart for the heart monitoring service and related requirements.

Platform Feature	Requirement
Wireless ECG device	Preferred
Local storage capability	Requested
Feedback on data acquisition	Mandatory
Battery life status notification	Requested
No user configuration steps	Mandatory
Broadband network interface	Preferred
Narrowband network interface	Mandatory
Wireless network connection	Preferred
Local storage capability	Mandatory
Data management	Asynchronous and Synchronous

**Table 7:** *Systems A*, *B*, and *C* technical ranking (*α* = 1, *β* = 0.8, and *γ* = 0.6).

System	no. of sensors (*N*)	S	CU	RP	TTP
*System A *	*N*_*A*_ = 7	*S*_*A*_ = 106	CU_*A*_ = 31	RP_*A*_ = 30	148.4
*System B *	*N*_*B*_ = 7	*S*_*B*_ = 109	CU_*B*_ = 31	RP_*B*_ = 42	159
*System C *	*N*_*C*_ = 6	*S*_*C*_ = 108	CU_*C*_ = 28	RP_*C*_ = 50	160.4
